# Engineered Human Nanoferritin Bearing the Drug Genz-644282 for Cancer Therapy

**DOI:** 10.3390/pharmaceutics12100992

**Published:** 2020-10-20

**Authors:** Elisabetta Falvo, Alessandro Arcovito, Giamaica Conti, Giuseppe Cipolla, Martina Pitea, Veronica Morea, Verena Damiani, Gianluca Sala, Giulio Fracasso, Pierpaolo Ceci

**Affiliations:** 1 CNR–National Research Council of Italy, Institute of Molecular Biology and Pathology, 00185 Rome, Italy; elisabetta.falvo@cnr.it (E.F.); veronica.morea@cnr.it (V.M.); 2 Dipartimento di Scienze Biotecnologiche di Base, Cliniche Intensivologiche e Perioperatorie, Università Cattolica del Sacro Cuore, Largo F. Vito 1, 00168 Rome, Italy; alessandro.arcovito@Unicatt.it; 3 Fondazione Policlinico Agostino Gemelli, IRCCS, 00168 Rome, Italy; 4 Department of Neurological and Movement Sciences, University of Verona, 37134 Verona, Italy; giamaica.conti@univr.it; 5 Thena Biotech, 04100 Latina, Italy; giuseppecipolla92@gmail.com; 6 Department of Biochemical Sciences, University Sapienza, 00185 Rome, Italy; martina.pitea@uniroma1.it; 7 Center for Advanced Studies and Technology (CAST), Department of Medical Oral and Biotechnological Sciences, University of Chieti-Pescara, 66100 Chieti, Italy; verena.damiani@unich.it (V.D.); g.sala@unich.it (G.S.); 8 Department of Medicine, University of Verona, 37134 Verona, Italy

**Keywords:** nanomedicine, human ferritin, gastrointestinal tumors, non-camptothecin topoisomerase I inhibitors, drug-delivery, CD71

## Abstract

Gastrointestinal tumors, including pancreatic and colorectal cancers, represent one of the greatest public health issues worldwide, leading to a million global deaths. Recent research demonstrated that the human heavy chain ferritin (HFt) can encapsulate different types of drugs in its cavity and can bind to its receptor, CD71, in several solid and hematological tumors, thus highlighting the potential use of ferritin for tumor-targeting therapies. Here, we describe the development and characterization of a novel nanomedicine based on the HFt that is named The-0504. In particular, this novel system is a nano-assembly comprising an engineered version of HFt that entraps about 80 molecules of a potent, wide-spectrum, non-camptothecin topoisomerase I inhibitor (Genz-644282). The-0504 can be produced by a standardized pre-industrial process as a pure and homogeneously formulated product with favourable lyophilization properties. The preliminary anticancer activity was evaluated in cultured cancer cells and in a mouse model of pancreatic cancer. Overall results reported here make The-0504 a candidate for further preclinical development against CD-71 expressing deadly tumors.

## 1. Introduction

Protein-cage molecules based on the H-chain of human ferritin (HFt) have been recently attracting growing interest in the field of cancer drug delivery, due to their excellent biocompatibility, selectivity for cancer over normal cells, binding to a large number of different human tumors and ability to encapsulate in their internal cavity high amounts (20–120 molecules) of different drug classes [1,2,3,4,5,6,7,8,9,10,11,12,13,14]. The cage-like shape is due to the fact that HFt is a multimeric protein consisting of 24 identical subunits that self-assembly into a symmetric hollow sphere, with external and internal diameters of 12 and 8 nm, respectively [15,16,17,18]. The physiological role of HFt is iron storage and delivery. As a carrier, the HFt system has several uniquely advantageous features: (a) encapsulated drug molecules are shieled from the external environment while in the bloodstream, thereby preventing payload leakage and off-target effects; (b) HFt is effectively taken up and rapidly internalized by virtually all types of cancer cells via the transferrin receptor 1 (TfR1, CD71), leading to massive intracellular protein accumulation [19,20,21,22]; and (c) selective pressure for iron uptake, which is necessary to sustain unrestrained cell growth, determines CD71 overexpression levels up to 100 times higher in the tumor as compared to normal cells. Thus, the HFt:CD71 nanocarrier:receptor system is very attractive for selective drug delivery, with one major limitation: like many proteins with ubiquitous housekeeping functions, CD71 is also expressed, although at low levels, on many dividing healthy cells, including hematopoietic progenitors.

To increase the selectivity of wild-type HFt for cancer cells over healthy ones, in the past few years we have developed two HFt genetic variants, named HFt-MP-PAS and HFt-MP-PASE [23,24]. In these variants, HFt is joined with a peptide sequence specifically cleavable by metalloproteases 2 and 9 (MP). The MP, in turn, is linked to masking polypeptides, named PAS or PASE, because they comprise only amino acids proline (P), alanine (A), serine (S), and in the case of PASE, glutamic acid (E). PAS and PASE polypeptides enhance HFt water-solubility and determine a 10/15-fold drop in CD71 binding affinity, as determined by surface plasmon resonance experiments [24]. Decreased binding to the bulk of CD71 molecules expressed by a multitude of normal tissues, albeit at low levels, both extends the half-life of the nanocarrier in the bloodstream as compared to native HFt [23,24,25,26] and increases the nanocarrier chances to recirculate until it binds at highly dense in CD71, but dimensionally small tumor site. Once there, the PASE-linked MP sequence is selectively and conditionally cleaved off by matrix metalloproteases 2 and 9, which are greatly enriched in the tumor microenvironment but essentially absent in normal tissues, leading to HFt unmasking and local restoration of HFt:CD71 binding and tumor killing. The HFt-MP-PASE construct proved indeed to be capable of delivering the canonical anticancer drugs doxorubicin and mitoxantrone (Mit), both of which were stably entrapped in the internal HFt cavity, to human pancreatic (PaCa44) and head and neck (FaDu) tumor xenotransplants in vivo, with good therapeutic efficacy [23,24,26]. However, complete and durable tumor regression could not be obtained either by us using these payloads, or by other groups using different HFt:drug nanosystems [2,23,26].

This prompted us to genetically modify our HFt-MP-PASE nanocarrier to load it with a different payload, named Genz-644282, which is both more potent and more versatile than Dox and Mit. Genz-644282 is a non-camptothecin topoisomerase I inhibitor that shows potent activities against a large number of human tumor cell lines, with IC50s ranging from 1.8 nM to 1.8 μM [27]. In addition, Genz-644282 is cytotoxic on camptothecin-resistant human cancer cell lines [28].

To allow Genz-644828 encapsulation, we designed a novel HFt-MP-PASE variant, named The-05, bearing ad hoc designed genetic point-mutations in the HFt internal surface. The-05 was actually able to effectively incorporate high amounts of Genz-644282, yielding the The-0504 nanocarrier:drug complex.

To investigate whether The-0504 was capable to kill cancer cells, we used it to treat different cancer cell lines in vitro and a pancreatic model in vivo in comparison with the free drug. 

## 2. Materials and Methods

### 2.1. The-05 Construct Design and Production

To effectively encapsulate the anti-cancer drug Genz-644282, which is expected to bear positive charges at physiological pH values, we designed a variant of the previously reported HFt-MP-PASE construct [24] with an enhanced negative charge of the HFt internal surface. To this end, we analyzed the three-dimensional (3D) structure of HFt which has been experimentally determined by X-ray crystallography [29] and is available from the Protein Data Bank archive (PDB ID: 1FHA). Structure visualization and analysis was performed with InsightII (Accelrys Inc., San Diego, CA, US) and PyMol. We chose to introduce the negatively charged glutamic acid residue (Glu) in place of four native HFt residues, namely Lys53, Lys71, Thr135, and Lys143. These residues were selected because they satisfy the following criteria: (i) their side-chains are solvent accessible on the internal surface of the HFt cavity; (ii) they are not involved in interactions, either within the monomer or at inter-monomer interfaces, whose alteration might undermine the stability of the tertiary structure or quaternary assembly, respectively; (iii) they are not involved in metal binding at the ferroxidase center of HFt; (iv) the three Lys side-chains are positively charged, and therefore their removal contributes to the generation of a negatively charged surface; (v) their relative distance in the 3D structure is high enough that Glu residues introduced at these positions are not expected to give rise to unfavorable interactions, either with their surrounding residues or with one another. Finally, structure superposition between mutated and wild-type HFt structures available from the PDB indicates that introduction of mutations at positions 53 (i.e., Lys53Cys, PDB ID: 4DZ0) and 143 (i.e., Lys143Cys, PDB IDs: 3ERZ, 2Z6M, 3ES3) does not determine detectable changes in either the monomer fold or multimeric assembly.

The variant of the HFt-MP-PASE construct bearing the Lys53Glu, Lys71Glu, Thr135Glu and Lys143Glu mutations was provided by Thena Biotech (Latina, Italy) and named The-05. It was obtained via recombinant protein technology, as previously reported. In brief, the expression vector pET-17b containing the *The-05* gene was assembled by GENEART AG (Germany). Gene synthesis was performed taking into account codon-optimization for high level expression in *Escherichia coli*. The recombinant protein The-05 was expressed in *E. coli*, purified and quantified as previously described [24].

### 2.2. The-0504 Production

Genz-644282 TFA salt (MedKoo Biosciences, Morrisville, NC, US) was encapsulated in The-05 using the ferritin disassembly/reassembly procedure previously described for other drugs resulting in a 120:1 molar ratio between the drug and The-05 protein [24]. The final product, named The-0504, was provided as lyophilized powder by Thena Biotech. Briefly, solutions of The-05 (2 mg/mL) in 15 mM NaCl was incubated for 10 min at pH 3.1 (pH adjusted with HCl). Protein disassembly/reassembly was achieved by dropwise addition of NaOH to pH = 7.5. After 20 min of stirring at room temperature, the product was filtered to eliminate insoluble particles. An excess of unbound drug was removed using 100 kDa Amicon Ultra-15 centrifugal devices, in 20 mM Tris-HCl at pH 7.5. Finally, the solution was sterile filtered and stored at 2–8 °C in the dark. The obtained system, made of The-05 containing Genz-644282 in the internal cavity, was named The-0504. Genz-644282 content of the samples was determined by UV-vis spectroscopy, after extracting the drug in 0.1 N HCl. Genz-644282 was quantified by using the calculated molar extinction coefficient ε = 10,000 M^−1^ cm^−1^ at 345 nm, with a linearity comprises between 0.25–0.45 AU. Protein content was also determined by UV-vis spectroscopy applying the following correction for the Absorbance at 280 nm: A_280nm_ − (A_345nm ×_ 4.2). The-0504 was stored as lyophilized powder at 2–8 °C and checked monthly for its stability. The-0504 production, formulation, and lyophilization process development studies were carried-out in collaboration with BSP pharmaceuticals (Latina, Italy).

### 2.3. Gel Electrophoresis (SDS-PAGE), Size-Exclusion Chromatography (SEC), Transmission Electron Microscopy (TEM) and Dynamic Light Scattering (DLS)

The purity of all the preparations was assessed by SDS-PAGE on 15% acrylamide gels stained with GelCode™ Blue Safe Protein Stain (Thermofisher scientific, Milan, Italy). SEC experiments were performed using a Superose 6 gel-filtration column equilibrated with phosphate buffered saline (PBS) at pH 7.4. Protein and drug contributions at 280 nm and 345 nm, respectively, were simultaneously followed.

In TEM experiments, an aliquot (8 µL) of empty (The-05) or drug-loaded (The-0504) protein (0.1 mg/mL) was dispersed on carbon coated glow discharged copper grids. Sample adsorption on carbon film was allowed for about 5 min, and sample excess was adsorbed with filter paper. Then, staining with 2% uranyl acetate solution was performed for about 30 s in the dark at room temperature; staining excess was adsorbed with filter paper and the grid washed with ultrapure water. Grids were then air dried for 1h before observation at room temperature. Transmission electron micrographs were collected working with a voltage of 120 keV at a magnification of 30,000× (Libra 120, Carl Zeiss AG, Oberkochen, Germany).

DLS experiments were carried out using a Zetasizer Nano S (Malvern Instruments, Malvern, UK) equipped with a 4 mW He–Ne laser (633 nm), as previously described [24]. Briefly, the measurements were performed at 25 °C, at an angle of 173° with respect to the incident beam. The average hydrodynamic diameters (Z-average diameter) of the scattering particles were calculated using peak intensity analyses. Results are the average of at least five measurements. Empty (The-05) or drug-loaded (The-0504) proteins were prepared at 1 mg/mL in diluted PBS (1:2).

All the traces for DLS experiments were analyzed with the software Origin 8.0 (Originlab Corporation, Northampton, MA, USA).

### 2.4. Antiproliferative Effects of The-0504 In Vitro

Human colorectal (HT29) and pancreatic (MiaPaCa2 and HPAF II) cells were obtained from American Type Culture Collection (Manassasas, VA, US); PaCa44 cells were kindly provided by Prof. A. Scarpa (Verona University, Verona, Italy).Cancer cells were passaged every 4 days using RPMI Medium. Media was supplemented with 2 mM glutamine, 10% of FBS and antibiotics. To perform the in vitro proliferation assays 5 × 10^3^ cells per well were plated in 96-well culture microplates. After overnight incubation, cells were treated in triplicate with 10 µL of serial dilutions of free Genz-644282 or The-0504 for 72 h. Then, the medium was replaced with fresh medium w/o phenol red supplemented with XTT (2,3-Bis-(2-Methoxy-4-Nitro-5-Sulfophenyl)-2H-Tetrazolium-5-Carboxanilide) reagent (Sigma-Aldrich, St Louis, MO, USA), according to manufacturer’s instructions. Finally, cells were incubated at 37 °C and the developed staining measured at 450 nm by a microplate reader (VERSAmax, Molecular Devices, Sunnyvale, CA, USA). The percentage of cell viability was estimated by comparing cells treated with free Genz-644282 or The-0504 to mock treated cells.

### 2.5. Therapeutic Evaluation of The-0504 In Vivo

Preliminary evaluation of The-0504 therapeutic activity was carried-out using human pancreatic HPAF II cancer cells. Female, 4–6-week-old CD1 nude mice (Charles River Laboratories; Calco, LC, Italy), were injected subcutaneously in the right flank with 3 × 10^6^ cells, previously resuspended in 200 μL of PBS. When subcutaneous tumors reached a volume of about 80-100 mm^3^, mice were randomized in groups of six animals and injected i.v. with 200 μL of PBS, Genz-644282 or The-0504. The-0504 powder provided by Thena Biotech was reconstituted in water about one hour before administration. Genz-644282 was formulated in sodium lactate buffer as previously reported [27]. The treatment dose normalized to Genz-644282 concentration was 1.5 mg/Kg. Mice were treated six times, twice a week for three weeks. Tumor volume was measured using a caliper. Mice were monitored for bodyweight and clinical signs. A tumor volume of about 1000–1500 mm^3^ was chosen as the endpoint after which mice were culled. Overall survival was also evaluated. The study period was set at 50 days from the beginning of therapy. 

Animal studies were performed according to the principles laid down in the European Community Council Directives (86/609/EEC) and in accordance with a protocol approved by the Institutional Animal Care and Use Committee of the University of Chieti and authorized by the Italian Ministry of Health (Protocol no. 457/2018-PR, 20 June 2018).

## 3. Results and Discussion

### 3.1. The-05 Variant of the HFt-MP-PASE Nanocarrier Was Generated and Characterized

**Figure 1:**
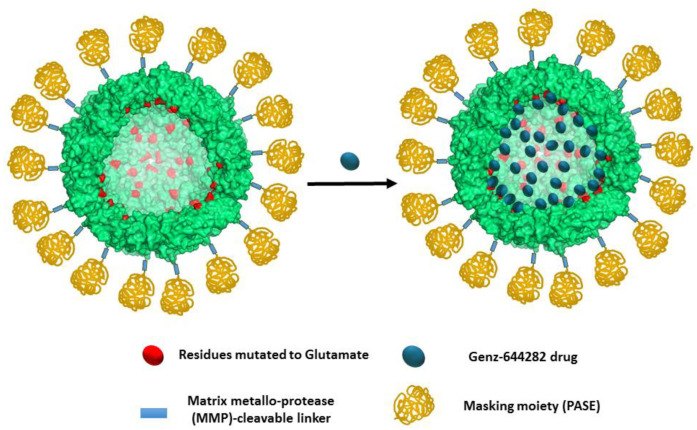
Schematic representation of The-0504. The Lys53, Lys71, Thr135 and Lys143 residues are mutated to Glutamate (Glu) in the The-05 protein (left). Mutated residues are colored red. Metalloprotease cleavable sequence and PASE polypeptide are colored light blue and gold, respectively. Genz-644282 drug is colored blue. All other residues are light green. To allow the internal surface of the protein to be visualized (lighter colors), only 18 monomers are shown. The picture has been generated with PyMol.

Our first aim in this study was to modify the previously reported cancer-selective HFt-MP-PASE nanocarrier to enable it to efficiently encapsulate the potent anti-cancer drug Genz-644282. Due to the presence of basic moieties in this drug, we decided to enhance the negative charge of the internal cavity of the HFt component of the nanocarrier. Based on the analysis of the three-dimensional (3D) HFt structure, which was experimentally determined by X-ray crystallography and is available from the Protein Data Bank (PDB) [29], we selected four native HFt residues, namely Lys53, Lys71, Thr135 and Lys143 to be mutated into negatively charged glutamic acid residues (Figure 1; see Methods section). The resulting variant, named The-05, was obtained via recombinant protein technology and purified from the cellular soluble fraction at high yield (about 150 mg/L of *E. coli* cell culture at lab scale; about 3 g/L at 10 L high-density fermentation scale).

**Figure 2:**
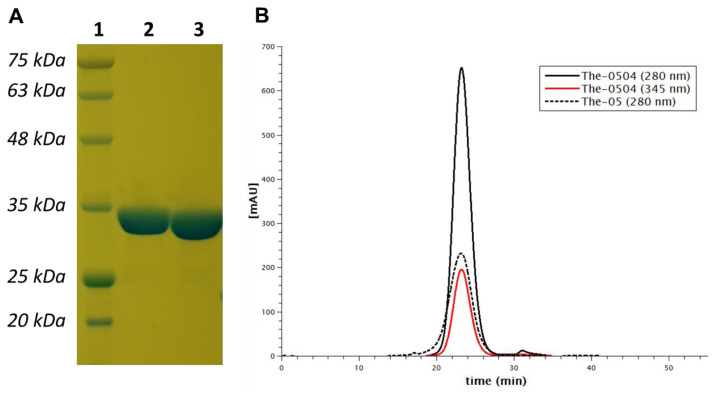
Purity of the The-05 nanocarrier before and after Genz-644282 encapsulation. (**A**) SDS-PAGE band migration profiles: Lane 1, protein marker; Lane 2, The-05 (10 µg); Lane 3, The-0504 (10 µg). (**B**) Size-exclusion chromatography analysis of The-05 and The-0504 detecting simultaneously The-05 protein and Genz-644282 contributions at 280 nm (black) and 345 nm (red), respectively.

**Figure 3:**
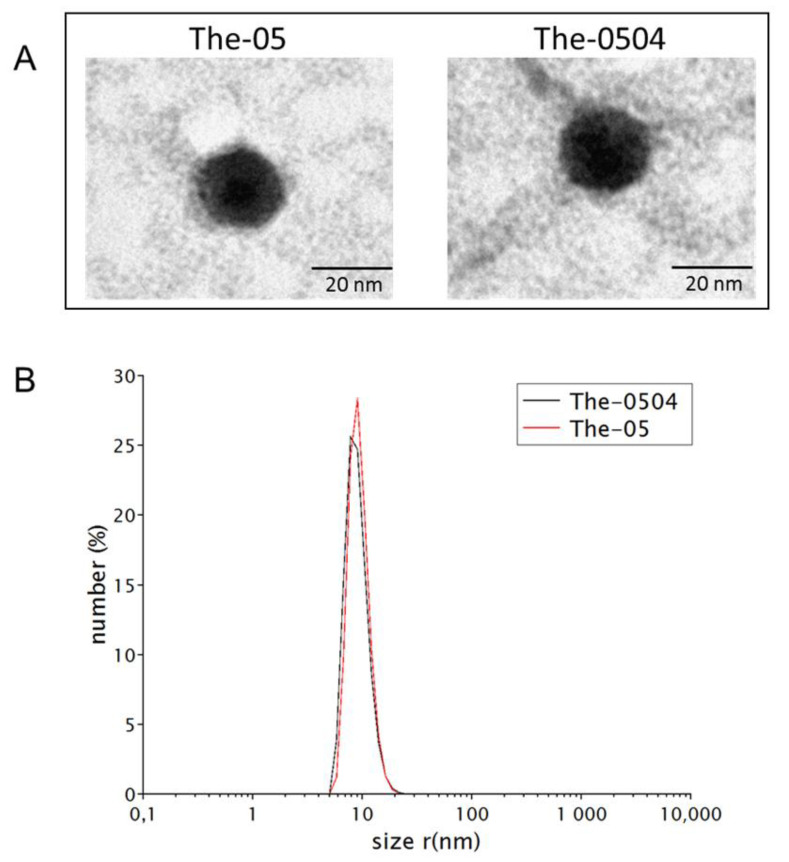
Biophysical characterization of the The-05 nanocarrier before and after Genz-644282 encapsulation. (**A**) Representative TEM images of negatively stained The-05 (left) and The-0504 (right), showing the spherical shape typical of ferritin-like proteins. (**B**) Dynamic light scattering profiles of The-05 (red) and The-0504 (black).

The purity, size, and overall assembly of The-05 construct were analyzed by gel electrophoresis (SDS-PAGE), size-exclusion chromatography (SEC), dynamic light scattering (DLS) and transmission electron microscopy (TEM) experiments. In SDS-PAGE gel electrophoresis, The-05 migrated at about 35 kDa as single pure band (Figure 2A). Overall, samples were found to be highly pure and monodispersed in solution (Figure 2B and Figure 3). The mean diameter of The-05 was about 18.5 nm as assessed by DLS (17.8 ± 0.6 nm) and TEM (18.8 ± 1.7 nm) analyses (Figure 3). Moreover, microscopy images revealed that the protein retains the ability of native human ferritin to adopt a spherical structure (Figure 3A).

### 3.2. The-05 Was Loaded with Genz-644282 and the (The-0504) Product Was Characterized

The non-camptothecin topoisomerase I inhibitor Genz-644282 was encapsulated inside the internal cavity of the novel The-05 construct by adapting the well-established protein disassembly/reassembly method (see Methods section). This procedure resulted in the encapsulation of about 80 molecules (81.0 ± 6.0) of the drug inside the The-05 cavity, with a protein recovery of about 70%. The nanocarrier:drug complex made by The-05 and the entrapped Genz-644282 was named The-0504 (Figure 1). By comparison, the original HFt-MP-PASE construct, which bears the native Lys53, Lys71, Thr135 and Lys143 residues on the internal surface, is significantly less effective since it encapsulates only about 15 (15.0 ± 2.1) Genz-644282 molecules, with a protein recovery of about 35%. To our knowledge, this is the first report describing the successful high-density encapsulation of the Genz-644282 drug in a nano-delivery system.

Sample polydispersity and overall assembly were assessed by SEC, DLS and TEM experiments. No significant differences between samples were observed before (The-05) and after (The-0504) Genz-644282 encapsulation, indicating that this process does not affect the overall protein structure and assembly (Figure 2). The mean diameters of The-0504 were (17.0 ± 0.7 nm) and TEM (19.7 ± 1.5 nm) as assessed by DLS and TEM analyses, respectively. In addition, the chromatography elution pattern (SEC) shows co-elution of the protein (280 nm) and drug (345 nm) moieties, proving the composite nature of the produced material (Figure 2B). SEC profiling did not highlight any evidence hinting at compound aggregation or drug loss following The-0504 formulation as lyophilized powder, storage at 2–8 °C for four months, and reconstitution in water (data not shown). Longer storage times are currently under evaluation.

### 3.3. The-0504 Shows High In Vitro Activity against Different Tumor Cell Lines

To assess the ability of Genz-644282-loaded The-0504 to kill cancer cells in vitro, we performed XTT viability assays on some human cancer cell lines of gastrointestinal origin: colorectal (HT29) and pancreatic (PaCa44, HPAF II and MiaPaCa2) cancer cells.

**Figure 4:**
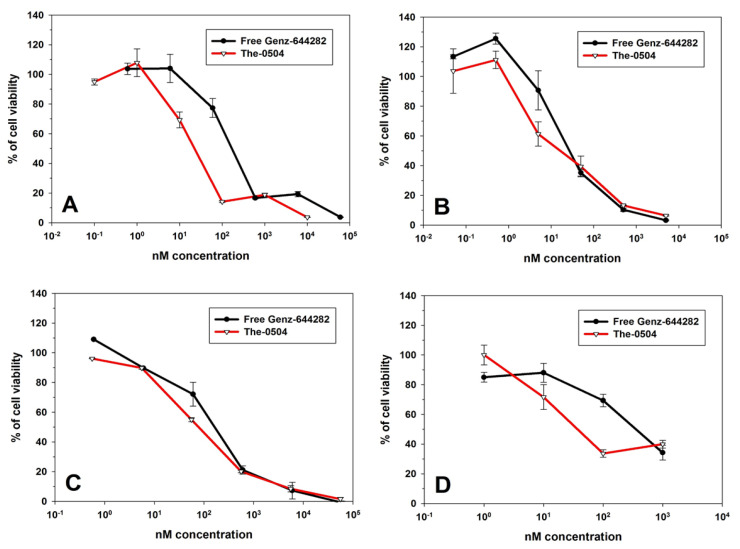
In vitro cytotoxicity. XTT assays performed on four cancer cell lines. (**A**) HT-29 colorectal cancer cells (**B**) MiaPaCa 2 pancreatic cancer cells; (**C**) PaCa44 pancreatic cancer cells and (**D**) HPAF II pancreatic cancer cells.

Results reported in Figure 4 indicate that Genz-644282 encapsulated within The-05 nanocarrier maintains its pharmacological activity and has a cytotoxic activity comparable or even superior to that of free Genz-644282 in all tested cell lines. The relative compound concentration yielding 50% cell viability (IC50, nM) of The-0504 and free Genz-644282 are the following: 21.0 ± 2.1 (The-0504) and 160.3 ± 20.5 (Genz-644282) for HT-29 cells; 14.5 ± 1.6 (The-0504) and 26.1 ± 2.6 (Genz-644282) for MiaPaCa 2 cells; 100.4 ± 19.3 (The-0504) and 150.4 ± 30.6 (Genz-644282) for PaCa44 cells; 35.9 ± 4.1 (The-0504) and 365.4 ± 58.0 (Genz-644282) for HPAF II cells. This is remarkable, since naked drugs can freely diffuse into cells, whereas The-0504 can only deliver the encapsulated Genz-644282 following rate-limiting receptor-mediated uptake. After these promising results, we decide to evaluate the preliminary antitumor activity of The-0504 in vivo.

### 3.4. The-0504 Is Highly Effective against a Xenograft Model of Pancreatic Cancer

**Figure 5:**
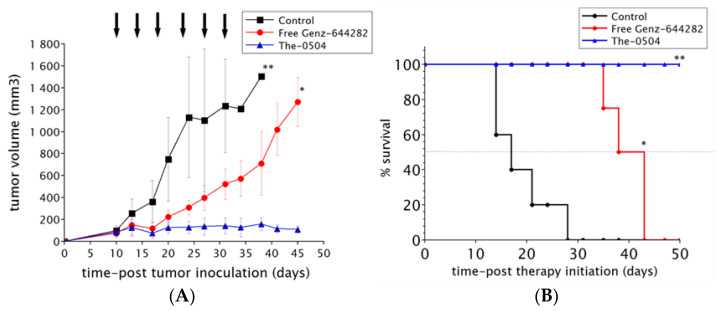
In vivo efficacy. (**A**) Tumor-growth curves for each mouse group are indicated. Student’s t-test is used to determine statistical significance. Control vs The-0504 and Control vs Free Genz-644282: ** *p* < 0.005; Free Genz-644282 vs The-0504: * *p* < 0.05. (**B**) Survival curves of different animal groups. Mice were sacrificed when the tumor had reached a volume in the range 1000–1500 mm^3^. Statistical analysis was performed by log-rank test. Control vs The-0504, and Control vs Free Genz-644282: ** *p* < 0.005; Free Genz-644282 vs The-0504 mg/Kg: * *p* < 0.05.

Efficacy of The-0504 was evaluated in a xenograft (subcutaneous) model of pancreatic (HPAF II cells) cancer. Tumor-bearing animals were randomized when the tumor was about 80–100 mm^3^ and treated with free Genz-644282 and The-0504, both at 1.5 mg/kg in Genz-644282, twice a week for three consecutive weeks by intravenous injections. As shown in Figure 5A, HPAF II tumor growth was significantly inhibited in mice treated with 1.5 mg/kg free Genz-644282; conversely, in control groups, tumors grew rapidly and reached a size >1000 mm^3^ at days 20–25 after tumor cell injection. However, tumor growth stalled for a short period of time, and resumed as soon as treatment was discontinued reaching a size >1000 mm^3^ at days 40. Tumor growth inhibition (TGI) value for free Genz-644282 was 53.3%. Strikingly, The-0504 showed a very high activity with a long-term regression of all established tumors. TGI value was 94.0%. Of note, 100% of mice treated with The-0504 were still alive at the end of the study period of 50 days from the beginning of therapy (Figure 5B), whereas the median survival of Genz-644282 treated-groups was 38.0 days. In addition, during the course of the experiment described above, mice were monitored for body weight and signs of pain or distress. No abnormal behavior or appreciable body-weight loss were observed in mice treated with The-0504.

## 4. Conclusions

We have designed and developed a novel HFt-based construct named The-05 characterized by the following features with respect to the native HFt protein: (a) outer masking (PASE) shell; (b) tumor-selective sequence responsive to proteolytic cleavage by MMPs; (c) 96 negatively charged glutamic acid residues inside the internal cavity for a better drug-binding. The-05 is expressed at high yields in *E. coli*; self-assembles into a wild-type like 24meric nanoparticle highly monodispersed; encapsulates stably up to 80 Genz-644282 molecules in the internal cavity. The-05 was named The-0504 after Genz-644282 encapsulation.

An in vitro assay demonstrated that The-0504 inhibited tumor cell proliferation with a slightly superior efficacy to the free drug Genz-644282. Strikingly, The-0504 displayed excellent therapeutic efficacy in a human pancreatic cancer model in vivo, increasing animal overall survivals significantly. The in vitro and in vivo properties of Genz644282-loaded construct fully justify carrying out additional preclinical studies (in progress) to assess its real potential for cancer therapy. 

## 5. Patents

P.C. and E.F. are inventors on patent application EP3186192B1 held by Thena Biotech that covers fusion proteins based on human ferritins and methods of use thereof.
